# The effect of discrete wavelengths of visible light on the developing murine embryo

**DOI:** 10.1007/s10815-022-02555-4

**Published:** 2022-06-23

**Authors:** Carl A. Campugan, Megan Lim, Darren J. X. Chow, Tiffany C. Y. Tan, Tong Li, Avishkar A. Saini, Antony Orth, Philipp Reineck, Erik P. Schartner, Jeremy G. Thompson, Kishan Dholakia, Kylie R. Dunning

**Affiliations:** 1 School of Biomedicine, Robinson Research Institute, The University of Adelaide, Adelaide, SA 5005 Australia; 2 Australian Research Council Centre of Excellence for Nanoscale BioPhotonics, The University of Adelaide, Adelaide, SA 5005 Australia; 3 Institute for Photonics and Advanced Sensing, The University of Adelaide, Adelaide, South Australia Australia; 4 National Research Council of Canada, Ottawa, Ontario Canada; 5 Australian Research Council Centre of Excellence for Nanoscale BioPhotonics, School of Science, Royal Melbourne Institute of Technology, Melbourne, VIC 3000 Australia; 6 School of Physical Sciences, The University of Adelaide, Adelaide, SA 5005 Australia; 7 Fertilis Pty Ltd, Adelaide, South Australia 5005 Australia; 8 School of Physics and Astronomy, University of St Andrews, North Haugh, Scotland , KY16 9SS UK; 9 School of Biological Sciences, The University of Adelaide, Adelaide, SA 5005 Australia; 10 Department of Physics, College of Science, Yonsei University, Seoul, 03722 South Korea

**Keywords:** Photodamage, Phototoxicity, Microscopy, Preimplantation embryo, Blastocyst

## Abstract

**Purpose:**

A current focus of the IVF field is non-invasive imaging of the embryo to quantify developmental potential. Such approaches use varying wavelengths to gain maximum biological information. The impact of irradiating the developing embryo with discrete wavelengths of light is not fully understood. Here, we assess the impact of a range of wavelengths on the developing embryo.

**Methods:**

Murine preimplantation embryos were exposed daily to wavelengths within the blue, green, yellow, and red spectral bands and compared to an unexposed control group. Development to blastocyst, DNA damage, and cell number/allocation to blastocyst cell lineages were assessed. For the longer wavelengths (yellow and red), pregnancy/fetal outcomes and the abundance of intracellular lipid were investigated.

**Results:**

Significantly fewer embryos developed to the blastocyst stage when exposed to the yellow wavelength. Elevated DNA damage was observed within embryos exposed to blue, green, or red wavelengths. There was no effect on blastocyst cell number/lineage allocation for all wavelengths except red, where there was a significant decrease in total cell number. Pregnancy rate was significantly reduced when embryos were irradiated with the red wavelength. Weight at weaning was significantly higher when embryos were exposed to yellow or red wavelengths. Lipid abundance was significantly elevated following exposure to the yellow wavelength.

**Conclusion:**

Our results demonstrate that the impact of light is wavelength-specific, with longer wavelengths also impacting the embryo. We also show that effects are energy-dependent. This data shows that damage is multifaceted and developmental rate alone may not fully reflect the impact of light exposure.

**Supplementary Information:**

The online version contains supplementary material available at 10.1007/s10815-022-02555-4.

## Introduction

Preimplantation embryo development is a highly sensitive period. During in vitro fertilization (IVF), preimplantation development is external from the oviduct, where its environment plays a critical role in development. Though mammalian embryos are capable of developing under varying culture conditions, sub-optimal conditions exert stressors that disrupt specific and global gene expression patterns [1, 2]. Oxygen level [3], temperature [4], pH [5], and culture media composition [6] have emerged as important culture factors which may determine the in vitro development of embryos. However, there is contention amongst other factors which may impact in vitro development including plastic-ware [7] and light exposure [8]. Though some studies suggest that embryos are exposed to some external light in vivo, this is far less than that present in vitro where light exposure is inevitable [9, 10].

In IVF clinics, light is used to observe embryos. In some cases, embryos are graded daily exposing embryos to light more frequently. A number of previous studies have investigated the impact of light on the developing embryo [11–18]. Light has several parameters that will affect embryos. These include wavelength, the average power applied, and the peak power of the light (if used in pulsed mode, e.g., multiphoton imaging)—all of which have to be considered with regard to the illuminated region and duration of illumination. Consideration of all these parameters contributes to the overall energy dose delivered to the embryo. The issue is further complicated in the previous literature as several papers use the measure of lux [11, 14, 15]. Lux is defined as one lumen per square meter (lm/m^2^), whereas irradiance is denoted in watts per square meter (W/m^2^) and is more commonly used for rigorous comparison. There is no direct conversion factor between lux and irradiance. This factor varies for each wavelength, and thus conversion is not straightforward unless one knows the spectral decomposition of the illumination source. As such, results employing lux as a measure are not as useful as those using irradiance [19]. Turning to irradiance itself, it is not just the power per unit area that matters but also (i) ensuring that this is applied uniformly across the whole embryo, (ii) the overall energy dose supplied which thus needs the period of irradiance to be considered, and (iii) knowledge of the spectral bandwidth of the light source. A key aspect of this present paper is to enable rigorous comparison of the effect between optical wavelengths by accounting for all the above-mentioned aspects.

Several previous studies have used broadband (often termed “white”) light and explored how that might affect the embryo [2, 8, 11–13, 16, 20]. In this context, broadband means the light source has a wide spectral bandwidth (> 50 nm). Takenaka et al. [16] compared “cool” versus “warm” fluorescent light, where the difference in source lies in the relative strengths of wavelength peaks around a wavelength of 430 nm (higher in “cool” light) versus those at 620 nm (higher in “warm” light). Umaoka et al. [20] exposed embryos to broadband fluorescence (from 340 to 760 nm) while Ottosen et al. [8] explored light exposure from a broadband source covering 400–700 nm. Bognar et al. [11] showed that exposure to broadband white light (400 to 700 nm, major peaks around 430 nm, 550 nm, and 620 nm) decreases implantation potential. This study took a step towards revealing wavelength selectivity by showing that restriction, largely to the red wavelength region (~ 620 nm), improved the implantation potential [11]. All these studies used lux to characterize the incident light. The use of filters restricted the optical emission but not to a level that this could be termed narrowband (≤ 10 nm). Overall, these studies indicated that there was an effect from light and that shorter wavelengths seemed more detrimental. While these studies broadly show that light may have an impact on embryo development, the precise influence on embryos in modern imaging methods requires a detailed study of narrowband light sources.

As label-free optical imaging to determine embryo developmental potential increase in popularity [21, 22], it is imperative that the impact of different wavelengths of light on the preimplantation embryo is carefully characterized. Mapping the stress tolerance embryos show for each wavelength may be advantageous in identifying how damage can be mitigated in clinical manipulation and modern imaging techniques. Our work aims to show the effect of wavelength during embryo development. In particular, the current study has direct relevance for the use of established and emerging optical microscopies such as confocal, multiphoton, hyperspectral, and fluorescence lifetime imaging which all often use narrow band illumination (wavelengths varying ± 10 nm around a given center wavelength). Here, we investigate the impact of four wavelengths with equivalent energy doses (blue (470 nm), green (520 nm), yellow (590 nm), and red (620 nm)) on the developing preimplantation embryo in vitro*.* We assess whether daily exposure during preimplantation development affects (1) development to the blastocyst stage; (2) levels of DNA damage, and (3) the number of cells within resultant blastocysts and their allocation to the inner cell mass. The generally considered benign wavelengths (yellow and red) were further investigated by assessing pregnancy and fetal outcomes following transfer to recipient females and assessing the impact of irradiance on intracellular lipid stores within the embryo.

## Methods

Unless otherwise stated, all chemicals were purchased from Sigma-Aldrich (St. Louis, MO, USA).

### Animals and ethics

Female (21–23 days old) and male (6–8 weeks old) CBA × C57BL/6 first filial (F1) generation (CBAF1) mice as well as female (6–8 weeks old) Swiss mice were obtained from Laboratory Animal Services (LAS; University of Adelaide, SA, Australia) and maintained on a 12 h light:12 h dark cycle. Animals were provided rodent chow and water ad libitum. All experiments were approved by the University of Adelaide Animal Ethics Committee (M-2019–052) and conducted in accordance with the Australian Code of Practice for the Care and Use of Animals for Scientific Purposes.

### Media for embryo handling and culture

Embryo handling and culture media were pre-equilibrated for 4 h at 37 °C in a humidified incubator of 5% O_2_, 6% CO_2_ with a balance of N_2_. Oviducts were collected in filtered Research Wash medium (ART Lab Solutions, SA, Australia) supplemented with 4 mg/mL low fatty acid bovine serum albumin (BSA, MP Biomedicals, AlbumiNZ, Auckland, NZ). Embryos were cultured in filtered Research Cleave medium (ART Lab Solutions, SA, Australia) supplemented with 4 mg/mL BSA.

### Collection of in vivo fertilized embryos and in vitro culture

Female CBAF1 mice were administered with an intraperitoneal (I.P.) injection of 5 IU equine chorionic gonadotrophin (eCG; Folligon, Braeside, VIC, Australia), followed by 5 IU of human chorionic gonadotrophin I.P. (hCG; Pregnyl, Kilsyth, VIC, Australia) 46 h later. Female mice were then mated with male mice of proven fertility. At 23 h post-hCG, females were culled via cervical dislocation, and oviducts dissected. Presumptive zygotes were harvested by puncturing the ampulla with a 29-gauge insulin needle. Presumptive zygotes were denuded using hyaluronidase (50 U/mL) diluted in in Research Wash medium for 2 min. Presumptive zygotes were then washed in Research Wash medium and screened for polar body extrusions to confirm successful fertilization. Zygotes were cultured within a 20 µL drop of Research Cleave medium overlaid with paraffin viscous oil (Merck Millipore, Darmstadt, Germany: 10 embryos per 20 µL; one centrally located drop per 35 mm dish). The size (4 mm) and positioning of the culture drops were standardized to reduce irradiance variation in embryo light exposure (< 10%; Supp Fig. 1). Embryos were cultured in vitro at 37 °C in a humidified incubator of 5% O_2_, 6% CO_2_ with a balance of N_2_.

### Exposure of developing preimplantation embryos to specific wavelengths of light using LEDs

To determine how visible light exposure impacts preimplantation embryo development, in vitro cultured embryos were irradiated with specific narrow-band wavelengths during development. On the day of exposure, culture dishes were removed from the incubator and placed on a 37 °C heating stage. Culture dishes were exposed to only one wavelength, while control, unexposed dishes were kept in the dark to limit ambient light exposure. Light-emitting diodes (LEDs) corresponding to blue (470 nm), green (520 nm), yellow (590 nm), and red (620 nm) wavelengths were placed under the culture dishes. Light from the LEDs passed through band pass filters (Thorlabs, NJ, USA), restricting the light to ± 10 nm around the center wavelength (Supp Fig. 1).

Light sources, including those on different microscopes, as well as different wavelengths, vary in power output. Duration is only one aspect of light exposure. For each of the narrow band light sources used in the current study, the power output was measured using an optical power meter (Thorlabs, NJ, USA) and calculated in Watts/cm^2^. An equivalent energy dose of 25.5 mJ/cm^2^ was calculated for each wavelength using the formula Time = Energy/Power (energy is in joules, power is in watts, and time is the seconds). By keeping the energy dose equivalent, it accurately quantifies the impact of the chosen wavelengths. To achieve this, the duration of exposure, per day, was calculated to be 17.2, 86.1, 96, and 26.7 s for the blue, green, yellow, and red wavelengths, respectively (Supp. Table 1). Importantly, duration was controlled for in all groups (including the unexposed control group) with all embryos spending equivalent duration outside the incubator. The energy dose used in the current study is broadly comparable with the dose used in other forms of microscopy such as confocal and multiphoton imaging or light microscopy used during standard IVF procedures in the laboratory [8, 23]. After LED exposure, all culture dishes were returned to the incubator and cultured in standard in vitro conditions until the following day of exposure. Following the last day of exposure, blastocyst-stage embryos were fixed and underwent immunohistochemistry for either γH2AX (DNA damage), OCT3/4 (allocation of cells to the inner cell mass), or staining with BODIPY (intracellular lipid).

### Assessment of on-time morphological development

Embryos were assessed for on-time morphological development on day 2 (2-cell; 46 h post-hCG) and day 5 (blastocyst stage; 118 h post-hCG). The rate of development to the 2-cell and blastocyst stages was calculated from the initial number of zygotes. Two-cell-stage embryos were identified by the presence of two regular blastomeres of equal size, while blastocysts were identified by the presence of a blastocoel cavity ≥ two-thirds the size of the embryo; or expanded; or hatching.

### Immunohistochemistry for DNA damage (γH2AX)

All immunostaining procedures were carried out at room temperature. Immunofluorescence for phosphorylated gamma-H2AX (γH2AX) was used to assess for double-stranded DNA breaks [24]. Blastocysts were fixed for 30 min in 200 µL of 4% paraformaldehyde (PFA) diluted in phosphate buffer saline (PBS). After fixation, embryos were washed with 200 µL of 0.3 mg/mL polyvinyl alcohol in PBS (PBV) and permeabilized for 30 min in 0.25% Triton-X in PBS. To prevent non-specific binding, embryos were blocked for 1 h in 10% goat serum (Jackson Immuno, Philadelphia, PA, USA) diluted in PBV. Embryos were then incubated for 24 h with anti-γH2AX rabbit monoclonal Alexa Fluor® 488-conjugated primary antibody (Ser139, 20E3, Cell Signaling Technology, Danvers, MA, USA) at 1:200 dilution in 10% goat serum. A negative control without primary antibody was also included. Following incubation, embryos were washed with PBV three times before incubation for 2 h in the dark with a goat anti-rabbit, Alexa Fluor® 594-conjugated secondary antibody (Life Technologies, Carlsbad, CA, USA) at 1:500 dilution in 10% goat serum. Embryos were also counterstained with 3 mM of 4,6-diamidino-2-phenylindole (DAPI) for 1 h in the dark to visualize nuclei. After secondary antibody incubation, embryos were washed with PBV three times and mounted on glass slides using DAKO mounting medium (Dako Inc., Carpinteria, CA, USA) before proceeding to imaging and analysis.

### Immunohistochemistry for the inner cell mass (OCT-3/4)

All immunostaining procedures were carried out at room temperature. Immunofluorescence for octamer-binding transcription factor-3/4 (OCT-3/4) was used to assess the number of cells within the inner cell mass lineage of the blastocyst-stage embryo. Embryos were fixed in PFA as described for γH2AX immunohistochemistry. After fixation, embryos were incubated with 0.1 M glycine at room temperature for 5 min and washed with PBV prior to permeabilization with 0.5% Triton X-100 for 30 min. Embryos were then blocked with 10% goat serum for 1 h prior to incubation in anti-OCT-3/4 mouse primary antibody for 24 h (Santa Cruz Biotech, Dallas, TX, USA) at 1:200 dilution in 10% goat serum. Following incubation, embryos were washed in PBV and incubated for 2 h in anti-mouse Alexa Fluor 488-conjugated secondary antibody (ThermoFisher, Waltham, MA, USA) at 1:500 dilution in 10% goat serum. Embryos were then counterstained with DAPI and mounted as described for γH2AX staining.

### BODIPY 493/503 staining

Blastocyst-stage embryos from unexposed and exposed groups (Supp. Table 2) were fixed in 4% paraformaldehyde-PBS for 30 min and rinsed thoroughly in PBV. Embryos were then incubated with BODIPY 493/503 (1 µg/mL; ThermoFisher) and DAPI (1.5 µM) in PBV for 1 h at room temperature in the dark. Embryos were thoroughly washed in PBV and mounted on glass slides in PBV before proceeding to imaging and analysis.

### Image acquisition and analysis

All images of γH2AX and OCT-3/4 immunostaining were captured on an Olympus FV3000 confocal laser scanning microscope (Olympus, Tokyo, Japan). Images were collected at 60 × magnification with an immersion oil compatible objective (Olympus, NA = 1.4). Images were captured at 4-µm intervals through the entire embryo and a final z-stack projection generated. Samples were excited at a laser wavelength of 405 nm (emission wavelength detection range: 430–470 nm) for DAPI, 594 nm (emission detection wavelength: 499–520 nm) for γH2AX, and 488 nm (emission detection wavelength: 490–525 nm) for OCT-3/4.

BODIPY 493/503-stained blastocysts were captured on an Olympus FluoView FV10i confocal laser scanning microscope (Olympus, Tokyo, Japan). Images were acquired at 60 × magnification with a water-immersion compatible objective (Olympus, NA = 1.2). Images were captured at 2-µm intervals through the entire embryo and a final z-stack projection generated. Samples were excited at 405 nm (emission wavelength detection range: 430–470 nm) and 488 nm (emission detection wavelength: 490–525 nm) to detect DAPI- and BODIPY-stained cells, respectively.

All image analysis was performed using ImageJ for Windows 10 (Fiji, MD, USA). For image analysis of γH2AX, z-stack images of DAPI and γH2AX were first merged, and then the number of nuclei containing γH2AX-positive foci counted manually. The number of inner cell mass (ICM) cells and total cell number (TCN) were quantified using OCT-3/4-positive and DAPI-stained cells, respectively. The percentage of ICM/TCN was also calculated for each blastocyst. Lipid abundance was quantified by fluorescence intensity of BODIPY staining in a z-stack projection for each embryo.

### Embryo vitrification and warming

To investigate the impact of light irradiation on subsequent pregnancy and post-natal outcomes, exposed and non-exposed embryos were vitrified and then warmed on the day of transfer. This was to ensure that both embryos and pseudopregnant females were at the developmentally appropriate stage on the day of transfer (blastocyst stage and 2.5 days post-coitum, respectively). For embryo vitrification, the base medium used for handling and vitrification was Research Wash medium (ART Lab Solutions, Australia). Handling medium consisted of Research Wash medium supplemented with 5 mg/mL low fatty acid bovine serum albumin (BSA, MP Biomedicals, AlbumiNZ, Auckland, NZ). The handling medium described above constituted the base for all embryo vitrification media.

The equilibration solution comprised of handling medium with 10% ethylene glycol and 10% dimethyl sulfoxide (DMSO). The vitrification solution comprised of 1 M sucrose dissolved in handling medium with 16.6% ethylene glycol and 16.6% DMSO. Warming solutions comprised of decreasing concentrations of sucrose (0.3 M, 0.25 M, and 0.15 M) diluted in handling medium. Embryos were warmed in Research Cleave medium (ART Lab Solutions, SA, Australia) supplemented with 4 mg/mL BSA.

Morula-stage embryos (96 h post-hCG) were vitrified with the CryoLogic vitrification method (CVM). A NUNC four-well dish (ThermoFisher Scientific, Waltham, MA, USA) was set up with 600 µL of handling medium, equilibration solution, and vitrification solution. Once media were warmed to 37 °C, embryos were rinsed twice in handling medium, followed by transfer into equilibration solution for 3 min. Embryos were then transferred into vitrification solution for 30 s and then loaded onto a Fibreplug (CryoLogic, Pty. Ltd, VIC, Australia). Once loaded, the Fibreplug was immediately vitrified in the vapor phase of liquid nitrogen, followed by storage in a Fibreplug straw within liquid nitrogen.

For embryo warming, 600 µL of handling medium supplemented with decreasing concentrations of sucrose (0.3, 0.25, and 0.15 M) was pre-warmed to 37 °C. Fibreplugs containing embryos were removed from their straws and immediately submerged in 0.3 M sucrose for 30 s, and then transferred into a well containing 0.25 M sucrose for 5 min. Next, embryos were transferred into 0.15 M sucrose for 5 min prior to incubation in handling medium for 5 min. Lastly, embryos were transferred into Research Cleave medium and cultured to the blastocyst stage. The post-warming survival rate was 80–85% for all groups (data not shown).

### Embryo transfer and postnatal outcomes

Blastocyst-stage embryos that were unexposed or exposed to yellow or red wavelength light were transferred into the uterine horns of pseudopregnant Swiss mice 2.5 days post-coitum. Embryo transfers were performed on mice under anesthesia with 1.5% isoflurane. Sixteen embryos were transferred per mouse, 8 embryos per uterine horn. Mice that underwent the embryo transfer procedure were monitored daily, with the number of pups from each female recipient recorded on delivery. At post-natal day 21, offspring were weighed and assessed for gross facial deformities.

### Statistical analysis

All statistical analyses were performed using GraphPad Prism version 9 for Windows 10 (GraphPad Holdings LLC, CA, USA). Data were checked for normality and appropriate statistical tests carried out as described in the figure legends. Proportional data were arcsine transformed prior to statistical analysis. *P* values < 0.05 indicated statistically significant differences.

## Results

The energy dose from optical microscopy can vary depending upon the exact type of imaging modality used. We chose a dose that was comparable with other forms of microscopy that are used during IVF treatments or for imaging the embryo to investigate the developmental and cellular impact of different wavelengths on the preimplantation embryo [8, 23].

### The impact of specific wavelengths on embryo development

**Fig. 1:**
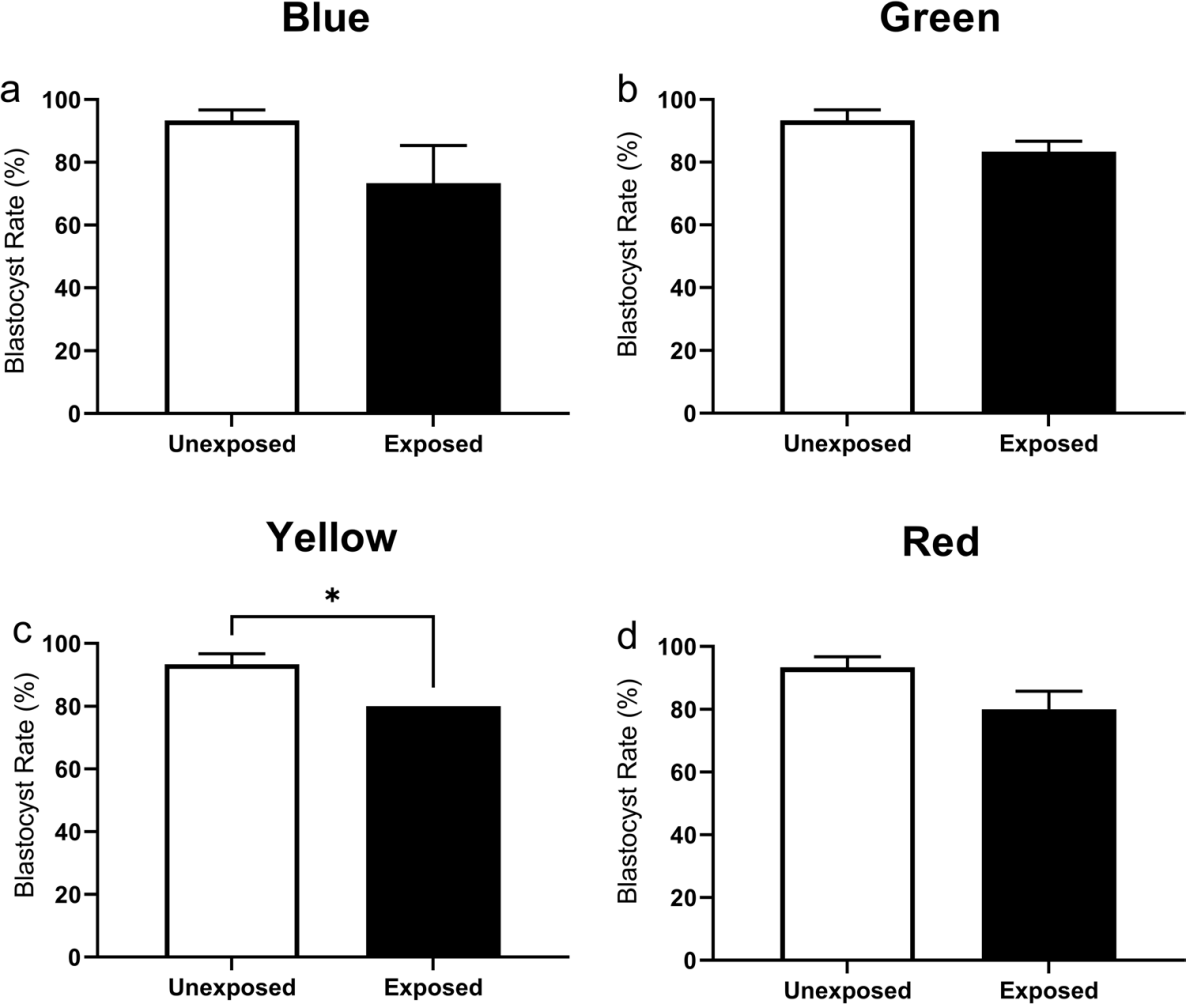
Exposure to yellow wavelength (590 nm) negatively impacted development to the blastocyst stage. Embryos were exposed daily to blue (**a**; 470 ± 10 nm), green (**b**; 520 ± 10 nm), yellow (**c**; 590 ± 10 nm), or red (**d**; 620 ± 10 nm) wavelengths during preimplantation development and compared to an unexposed control group. Blastocyst rate was calculated from the starting number of zygotes. Data are presented as mean ± SEM, from 3 independent experimental replicates; *n* = 21–28 embryos per group. Data were analyzed using a Mann–Whitney test (**a**, **b**, and **d**) or unpaired *t*-test (**c**). *P < 0.05

To assess whether exposure to varying wavelengths of light inhibits preimplantation embryo development, we first determined whether development to the 2-cell stage was affected following exposure at the 1-cell stage. Compared to the unexposed control group, no significant difference was observed in the 2-cell cleavage rates for blue (470 ± 10 nm), green (520 ± 10 nm), yellow (590 ± 10 nm), or red (620 ± 10 nm) wavelength exposed embryos (Supp. Fig. 2; *P* > 0.05). Similarly, there was no observable effect on development to the blastocyst stage when embryos were exposed blue, green, or red wavelengths daily (Fig. 1a, b, d, respectively; *P* > 0.05). In contrast, exposure to yellow wavelength resulted in significantly fewer embryos reaching the blastocyst stage of development compared to unexposed embryos (Fig. 1c; *P* < 0.05).

### The impact of specific wavelengths on DNA integrity within the developing embryo

**Fig. 2:**
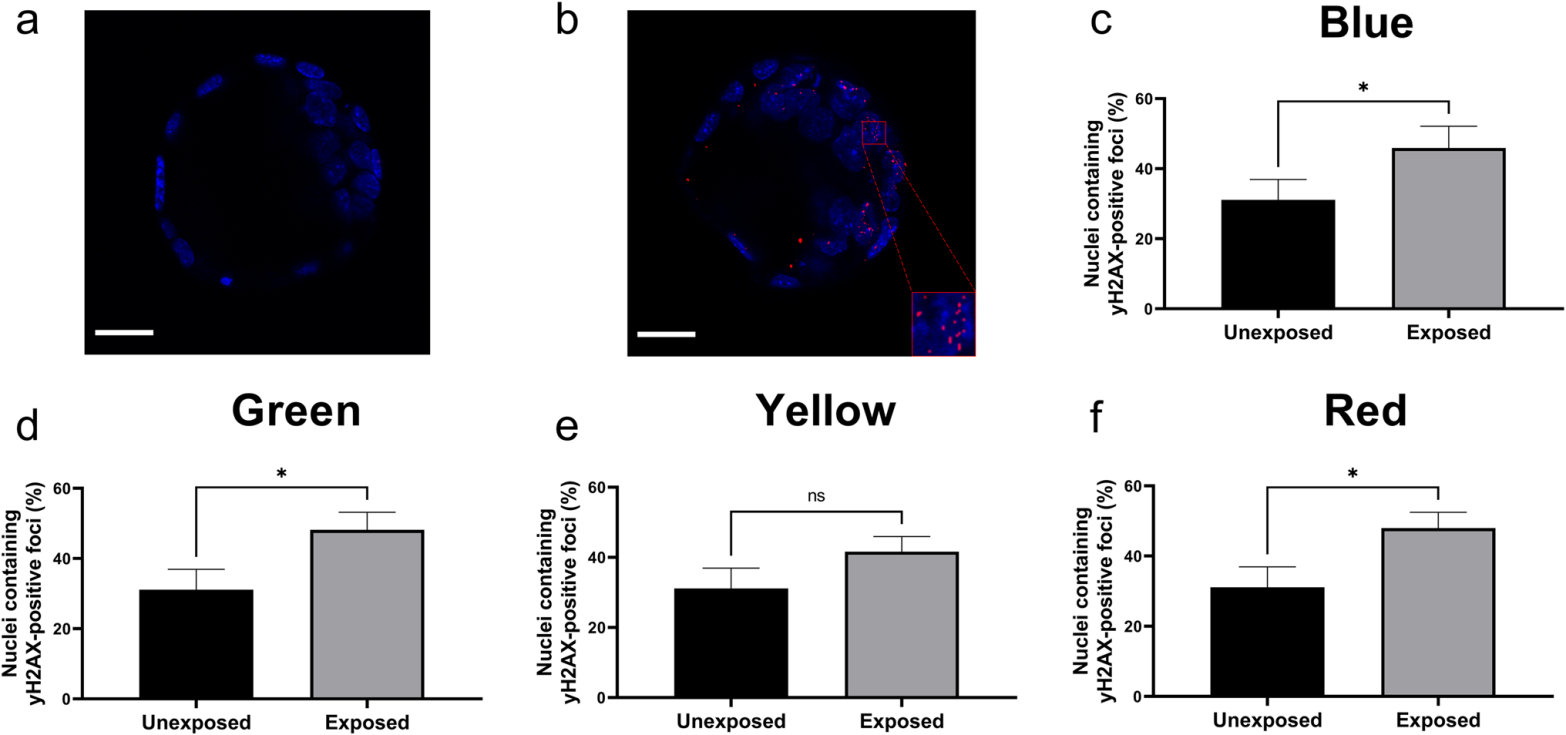
Exposure to blue (470 nm), green (520 nm), or red (620 nm) wavelength led to significantly increased DNA damage within resultant blastocyst. Double-stranded DNA damage in unexposed (**a**) vs exposed (**b**) blastocyst-stage embryos was assessed using γH2AX immunohistochemistry. Inset in (**b**) shows multiple γH2AX-positive puncta within a nucleus. Percentage of nuclei containing γH2AX-positive punctum was quantified within blastocysts following exposure to blue (**c**; 470 ± 10 nm), green (**d**; 520 ± 10 nm), yellow (**e**; 590 ± 10 nm), or red (**f**; 620 ± 10 nm) wavelengths during preimplantation embryo development. These were compared to an unexposed control group. Data are presented as mean ± SEM, from 3 independent experimental replicates, *n* = 9–16 embryos per group. Data were analyzed using a Mann–Whitney test. *P < 0.05. Scale bar = 25 µm

We next sought to determine whether irradiation with specific wavelengths during preimplantation development affected the level of DNA damage within resultant blastocyst-stage embryos (Fig. 2a, b). When compared to the unexposed control, we observed a significantly higher levels of DNA damage within blastocysts following daily exposure to blue, green, or red wavelengths (Fig. 2c, d, and f, respectively; *P* < 0.05). In contrast, exposure to the yellow wavelength during preimplantation development did not affect the level of DNA damage compared to the unexposed control group (Fig. 2e; *P* > 0.05).

### The effect of specific wavelengths on the number of cells and allocation to the inner cell mass within resultant blastocyst-stage embryos

**Fig. 3:**
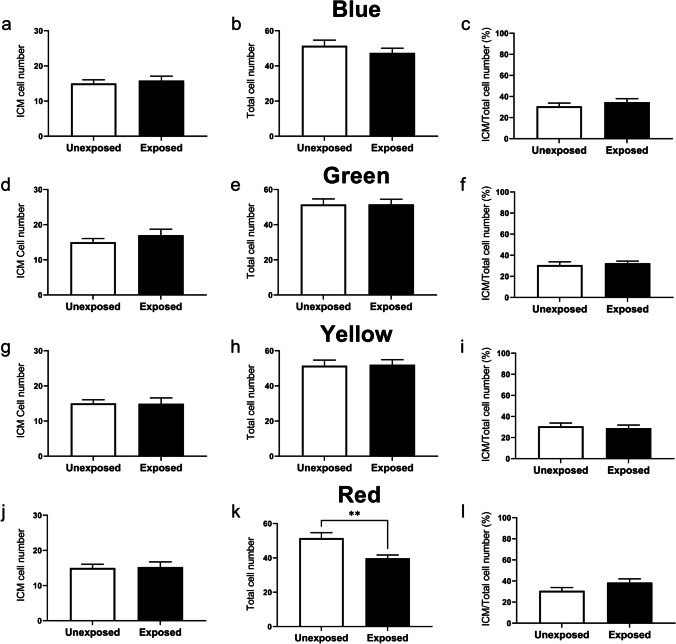
Exposure to red wavelength (620 nm) during preimplantation development significantly reduced total cell number within resultant blastocysts. The impact of wavelength-specific exposure on the number of cells within the inner cell mass (ICM; **a**, **d**, **g**, **j**), the total cell number (TCN; **b**, **e**, **h**, **k**), and the ratio of ICM/TCN (expressed as a percentage; **c**, **f**, **i**, **l**) of resultant blastocyst was assessed using Oct-3/4 (ICM) and DAPI (TCN). Embryos were either unexposed or exposed daily to blue (**a**, **b**, **c**; 470 ± 10 nm), green (**d**, **e**, **f**; 520 ± 10 nm), yellow (**g**, **h**, **i**; 590 ± 10 nm), or red (**j**, **k**, **l**; 620 ± 10 nm) wavelengths. Data are presented as mean ± SEM, from 3 independent experimental replicates; *n* = 11–13 embryos per group. Data were analyzed using a two-tailed unpaired *t*-test. **P < 0.01

To further characterize the impact of specific wavelengths on the developing preimplantation embryo, we quantified the number of inner cell mass (ICM) cells and the total cell number (TCN) in blastocyst-stage embryos. There was no impact on either the total cell number or allocation to the inner cell mass when embryos were exposed to the blue (Fig. 3a–c), green (Fig. 3d–e), or yellow (Fig. 3g–h) wavelengths compared to the unexposed control group. Interestingly, exposure to the red wavelength every day of preimplantation development resulted in blastocyst-stage embryos with significantly fewer cells, but comparable number of inner cell mass cells compared to unexposed embryos (Fig. 3j and k). This difference did not impact the inner cell mass/total cell number ratio (Fig. 3l).

### The impact of longer wavelengths on pregnancy rate and post-natal outcomes

**Fig. 4:**
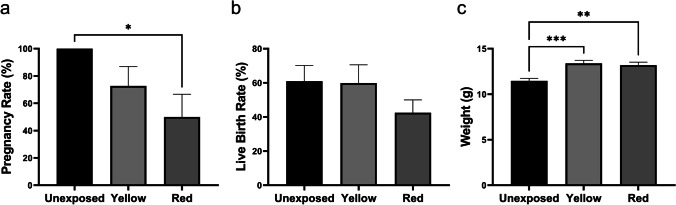
Exposure to longer wavelengths during preimplantation development reduces pregnancy rate (red; 620 nm) and leads to significantly higher weights at weaning (red and yellow; 590). The effect of red and yellow wavelengths on pregnancy rate (**a**), live birth rate (**b**), and the weight of offspring at weaning (**c**) was assessed following embryo transfer of blastocyst-stage embryos to pseudopregnant mice. Data are presented as mean ± SEM, *n* = 9–11 pseudopregnant females per group for pregnancy rate, *n* = 35–58 pups per group for live birth rate, *n* = 18–35 pups for weight at weaning. Normally distributed data were analyzed using a one-way ANOVA with Holm-Šídák post hoc test (**a**). A Kruskal–Wallis with Dunn’s multiple comparisons test was applied to data which did not follow a normal distribution (**b**). *P < 0.05, **P < 0.01, ***P < 0.001

It is generally accepted that longer wavelengths are safe for the developing preimplantation embryo [13, 14]. As exposure to longer wavelengths in the current study led to decreased numbers of embryos reaching the blastocyst stage (yellow), increased levels of DNA damage (yellow and red), and fewer cells within the blastocyst (red), we further explored whether these wavelengths impacted pregnancy success or post-natal outcomes. Following transfer to recipient females, there was a significant reduction in pregnancy rate when embryos were exposed to the red wavelength compared to unexposed control embryos (Fig. 4a; *P* < 0.05). In contrast, exposure to the yellow wavelength during preimplantation development did not affect pregnancy rate (Fig. 4a; *P* > 0.05). Exposure of embryos to either yellow or red wavelengths did not affect live birth rate compared to the unexposed control (Fig. 4b; *P* > 0.05). Interestingly, exposure to either yellow or red wavelengths during preimplantation development led to a significant increase in body weight at weaning compared to pups derived from unexposed control embryos (Fig. 4c; *P* < 0.01; Supp. Table 3). No gross facial deformities were observed in any treatment group.

### The effect of longer wavelengths on lipid abundance within resultant blastocyst-stage embryos

**Fig. 5:**
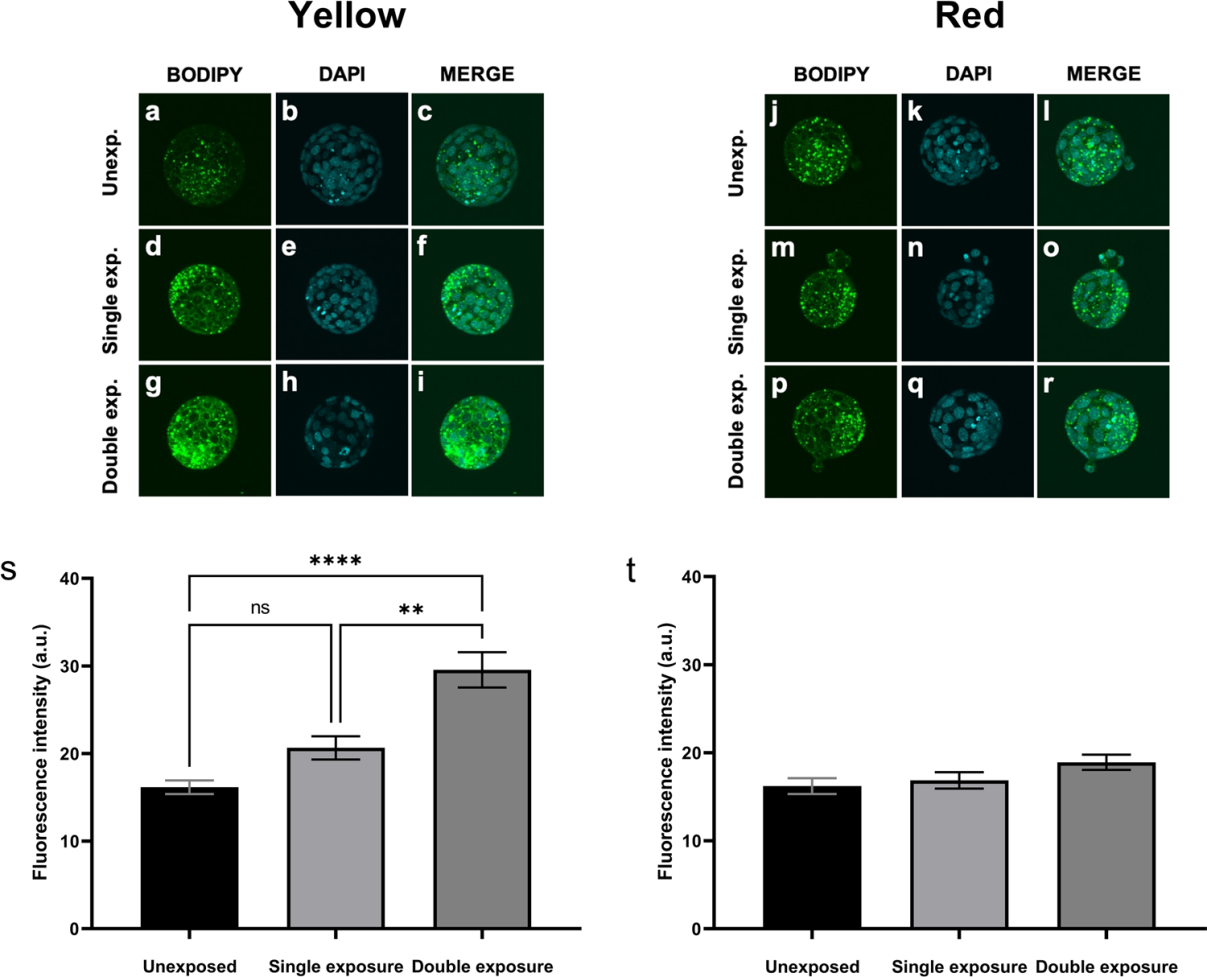
Yellow wavelength (590 nm) exposure during preimplantation development significantly increased lipid abundance within resultant blastocysts. Lipid abundance in blastocyst-stage embryos was assessed using BODIPY 493/503. Embryos were either unexposed (**a**–**c**; **j**–**l**) or exposed to yellow (**d**–**i**; 590 ± 10 nm) or red (**m**–**r**; 620 ± 10 nm) wavelengths during preimplantation embryo development. Exposed embryos were irradiated for a single (**d**–**f**; **m**–**o**) or double dose (**g**–**i**; **p**–**r**; see Supp. Table 2). Fluorescence intensity was quantified for embryos that were unexposed or exposed to yellow (**s**) or red (**t**) wavelength exposed embryos. Data are presented as mean ± SEM, from 4 independent experimental replicates, *n* = 26–37 embryos per group. Data were analyzed using a Kruskal–Wallis with Dunn’s multiple comparisons test. Images were captured at × 60 magnification. **P < 0.01; ****P < 0.0001

Previous work has shown that exposure of adipocytes to yellow or red wavelengths reduces intracellular lipid via lipolysis [25]. Lipids form an important energy source for the embryo, but an excess of lipid is damaging to developmental competence [26–28]. Thus, to investigate the mechanism by which longer wavelengths elicited a negative impact on the developing embryo, we quantified the abundance of intracellular lipid. Additionally, we explored whether any impact on lipid abundance was dose-dependent by exposing embryos to the same energy used in the experiments described above or to double the energy (Supp. Table 2). There was a visible increase in lipid abundance within embryos that were exposed to the yellow wavelength compared to unexposed controls (Fig. 5a-i). In contrast, there was no observable difference in lipid abundance when embryos were exposed to the red wavelength (Fig. 5j-r). The observable and contrasting impact of yellow and red wavelengths on lipid abundance were confirmed following quantification. Compared to unexposed embryos, there was a 1.3-fold increase in lipid abundance in embryos exposed to the yellow wavelength daily, although this did not reach statistical significance (Fig. 5s; single exposure vs unexposed). When exposure to the yellow light was doubled, there was a significant 1.8-fold increase in lipid abundance compared to the unexposed control group (Fig. 5s; double exposure vs unexposed; *P* < 0.0001). In contrast, exposure to the red wavelength did not affect levels of intracellular lipid compared to unexposed control embryos (Fig. 5t).

## Discussion

There has been an increase in popularity in using various forms of optical imaging to study the preimplantation embryo both from a clinical and a research standpoint. Such studies expose embryos to light, varying in intensity and wavelength [9, 10, 17]. This, however, may have damaging effects on the embryo [2, 12]. Previous investigations with embryos either focus on specific wavelength ranges [13, 14] or use broadband light sources [2, 8, 11–13, 16, 20], but there is no detailed consideration of the uniformity of illumination across all embryos and an absence of controlling the energy dose given for each wavelength range. In the current study, we address these shortcomings of previous work and conduct a thorough examination by exposing developing preimplantation murine embryos to specific wavelengths of light and controlled energy doses. We assess the impact on embryo viability, DNA damage, pregnancy/fetal outcomes, and the abundance of intracellular lipid. Therefore—as we discuss below—it is perhaps not too surprising that our conclusions contrast at times with previous literature. We contend that our approach adds key new knowledge to this burgeoning area and our conclusions are supported by additional analyses that reinforce our outcomes.

Light-induced damage on the developing preimplantation embryo has been the focus of previous studies. However, most of these have described irradiance intensity as lux only [2, 13–15, 20]. This has made direct comparison of results within and between studies challenging, and at times imprecise. In the current study, light was measured as a function of intensity and area. To accurately characterize wavelength-specific damage, we implemented an experimental design which accounted for irradiance intensity variation along the beam width by housing embryos within a 4 mm diameter drop of culture media where intensity variation was ≤ 10%. Additionally, a band pass filter was used to attenuate light to frequencies ± 10 nm around the center wavelength. Furthermore, as all wavelengths exert varying energy doses upon exposure, our experimental design tailored both the time and uniformity of exposure to ensure that the energy exposed to embryos was equivalent for each wavelength—a variable not accounted for in previous studies.

Across all wavelengths, only embryos exposed to the yellow wavelength (590 nm) daily had a significantly lower blastocyst rate compared to unexposed control embryos. To the best of our knowledge, our study is the first to show a negative impact of a long wavelength, rather than short [14], on development to the blastocyst stage. A previous study using differentiated adipocytes showed that amber light (590 nm) led to increased breakdown of lipid droplets [29]. In the current study, we investigated whether yellow light had a similar impact on the embryo. In contrast with the effect on adipocytes, exposure of embryos to the yellow wavelength led to an increase in lipid abundance. We also showed that the effect of the yellow wavelength was dose-dependent with delivery of increasing levels of energy correlating with increasing lipid within the embryo. Although lipid droplets have been found to be crucial for preimplantation embryo development, overabundance is harmful [28] which may explain the decreased blastocyst rate following irradiation with the yellow wavelength. Elevated levels of intracellular lipid within the embryo may have also contributed to the higher weight at weaning observed following transfer of yellow wavelength exposed embryos. The mechanism by which the yellow wavelength led to elevated lipid abundance and a higher weaning weight requires further investigation.

Analysis of γH2AX phosphorylation in blastocyst-stage embryos revealed unique impacts of specific wavelengths on DNA integrity. Embryos exposed daily to the blue, green, or red wavelengths had significantly higher levels of DNA damage. The effect of the blue wavelength is supported by a previous study showing elevated DNA damage following light (470 nm) exposure [2]. Intriguingly, however, are our outcomes for the red wavelength which contrast with previous work. Elevated DNA damage outcomes for red wavelength light were unexpected as a previous study showed lower hydrogen peroxide levels and HSP70 protein abundance after light (620–750 nm) exposure [14]. In the clinical setting, this outcome contrasts the claims of some time-lapse imaging systems that utilize red wavelength light to bypass the detrimental effects of short wavelength light [13, 30]. The negative effect of the blue and green wavelengths, resulting in elevated DNA damage, concurs with previous work where the same wavelengths led to increased reactive oxygen species (ROS) formation and HSP70 expression [12, 14], both indicators of cellular stress. Although certain wavelengths appeared more damaging to DNA, caution should still be exercised when using any visible light wavelengths.

Inner cell mass and trophectoderm populations at the blastocyst stage are predictive of pregnancy and live birth outcomes [31–33]. In this study, we showed no impact on the ICM or total cell number for blastocysts exposed to the blue, green, and yellow wavelengths. For blue wavelength light, our observations are inconsistent with previous findings [14], where the authors observed increased incidence of cell apoptosis. Though ICM numbers were comparable with unexposed embryos, TE numbers may have declined following red wavelength exposure as observed by significantly lower total cell number. This contrasts with earlier studies, where exposure of developing embryos to red wavelength light (620–750 nm) did not impact ICM, TE, or total cell numbers [13, 14]. Although our findings suggest no wavelength-specific impact on cell counts for blue, green, and yellow wavelength exposed embryos, caution should still be exercised as our DNA damage results may suggest increased ROS formation which may promote cell apoptosis [34].

Unique to the longer wavelengths was the negative effect of red wavelength exposure on pregnancy rate. This finding suggests that implantation was significantly impaired in red wavelength exposed embryos—an outcome that may be associated with the observed reduction in TE cells. In contrast, lipid droplet abundance was similar in red wavelength exposed groups relative to unexposed embryos. This result contrasts with the previous study on differentiated adipocytes where intracellular lipid was decreased following irradiation with a red wavelength (660 nm [25]). This suggests a difference in wavelength-specific tolerance between cells—an outcome alluded to in previous studies [8, 14, 19]. Further investigation into the mechanisms underlying these effects of the red wavelength on the preimplantation embryo is warranted.

Also pertinent to this present paper, a previous study showed that following spinning disk confocal imaging there was no impact on embryo development but the authors acknowledge that they did not use laser illumination and used very short exposure times [35]. Closest to the present work is the study by Squirrell et al. [23] who studied the dynamics of mitochondrial distribution in hamster embryos over 24 h using two-photon microscopy (operating at a wavelength of 1047 nm). This study showed that imaging did not affect development to blastocyst nor fetal developmental following transfer. In contrast, their study showed that confocal imaging (at wavelengths of 514 nm, 532 nm, and 568 nm) for only 8 h inhibits development. However, it is to be noted that this study explored labeled samples and worked on three narrow band, closely spaced wavelengths in the blue to green range of the spectrum.

Our study thus adds significant value to the field as we concentrate on the effects on embryo development upon irradiation of narrowband (± 10 nm) light centered around the blue (470 nm), green (520 nm), yellow (590 nm), and red (620 nm) regions of the spectrum. Importantly, this widely varying range of wavelengths captures some of the key ones used in confocal imaging as well as being aligned to other more advanced forms of optical imaging such as fluorescence lifetime studies, hyperspectral imaging, and light sheet microscopy. This is coupled with the fact that our study is carefully designed to ensure equivalent energy dose for all samples regardless of wavelength used, a feature not seen in previous studies. This makes our study robust and highly informative to users of such imaging modalities for embryo analysis. It is, however, important to note that the murine embryo was used as a model in the current study. Thus, any direct correlation with human embryos based on these results warrants caution and requires further investigation.

In the current study, we observed that the yellow wavelength led to increased lipid abundance within the blastocyst-stage embryo which may explain the decreased rate of development to the blastocyst stage and heavier weight of resultant offspring at weaning. Furthermore, in exposing embryos to the red wavelength we observed higher levels of DNA damage at the blastocyst stage which may explain the reduced number of trophectoderm cells and the lower pregnancy rate post-transfer. These findings suggest that wavelength-specific impact is multifaceted and analyses of embryo health after light exposure require both spatial (i.e., development to the blastocyst stage and pregnancy/post-natal outcomes) and molecular (i.e., DNA damage; lipid abundance) assessments. Our findings will inform future studies of the potential damage of visible light, particularly those in the long wavelength spectrum. Further investigations into the source of cell damage and the mechanisms underlying effects on lipid as well as pregnancy and fetal weights will aid in understanding the implications of this research to clinical embryology.

## Supplementary Information

Below is the link to the electronic supplementary material.

Supplementary file1 (DOCX 1.09 MB)

Below is the link to the electronic supplementary material.

Supplementary file2 (DOCX 33.2 KB)

## Data Availability

All data generated or analyzed during this study are included in this published article and are available from the corresponding author on reasonable request.

## Code availability

N/A.
