# Supplementary material for: The effect of discrete wavelengths of visible light on the developing murine embryo
Source: J Assist Reprod Genet. 2022 Jun 23;39(8):1825–37. doi: 10.1007/s10815-022-02555-4 (PMC9428105; doi:10.1007/s10815-022-02555-4)
Supplement: Supplementary file 1 — Supplementary file1 (DOCX 1.09 MB) [file 10815_2022_2555_MOESM1_ESM.docx]

Supplementary Figure 1. **Schematic of experimental design for LED light exposure.** Embryos (10) were cultured in a single drop of medium (20 µl, 4 mm in diameter) centered on a culture dish. Accounting for optical field heterogeneity from the LED source, the size of the drop was controlled to minimize the variation of intensity to < 10%. Each drop was overlaid with paraffin oil to prevent osmolality changes attributed to evaporation. The air gap (~ 10 mm) ensured that the LED uniformly illuminated the drop and thus embryos. Light from each LED passed through a bandpass filter to attenuate light frequencies to ± 10nm. *Schematic not to scale.


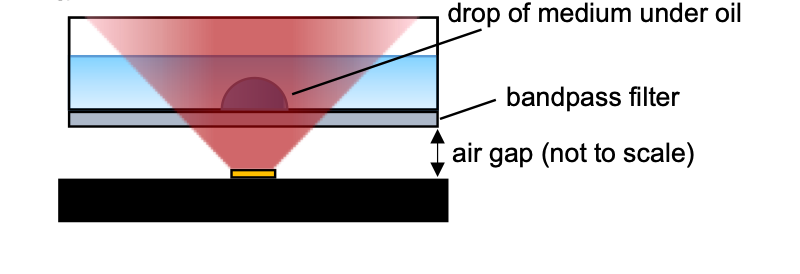


Supplementary Figure 2. **No impact on embryo cleavage rates following exposure at the 1-cell stage to (a) blue, (b) green, (c) yellow, and (d) red wavelengths.** Data are presented as mean ± SEM, from 3 independent experimental replicates; *n* = 26-29 embryos per group. Data were analyzed using a Mann-Whitney test.
