# Supplementary material for: The effect of discrete wavelengths of visible light on the developing murine embryo
Source: J Assist Reprod Genet. 2022 Jun 23;39(8):1825–37. doi: 10.1007/s10815-022-02555-4 (PMC9428105; doi:10.1007/s10815-022-02555-4)
Supplement: Supplementary file 2 — Supplementary file2 (DOCX 33.2 KB) [file 10815_2022_2555_MOESM2_ESM.docx]

Supplementary Table 1. Duration of exposure for each wavelength to achieve uniform energy dose of 25.5 mJ/cm^2^.

| LED (wavelength) | Power (mW/cm^2^) | Duration in secs |
| --- | --- | --- |
| Blue (470 nm) | 1.482 | 17.2 |
| Green (520 nm) | 0.296 | 86.1 |
| Yellow (590 nm) | 0.266 | 96.0 |
| Red (620 nm) | 0.955 | 26.7 |

Supplementary Table 2. Duration of exposure for yellow and red wavelengths to achieve a uniform energy dose of 25.5 mJ/cm^2^ (single exposure) or 51 mJ/cm^2^ (double exposure).

| LED (wavelength) | Power (mW/cm^2^) | Single exposure duration  (sec) | Double exposure duration  (sec) |
| --- | --- | --- | --- |
| Yellow (590 nm) | 0.266 | 96.0 | 192 |
| Red (620 nm) | 0.955 | 26.7 | 53.4 |

**Supplementary Table 3. Individual weight (g) of pups at weaning.** Pups derived from blastocyst-stage embryos that were either unexposed, or exposed to red or yellow wavelength during preimplantation development.

|  | Weight of pups at weaning (g) | | |
| --- | --- | --- | --- |
| Pup number | Unexposed | Yellow | Red |
| 1 | 13 | 12 | 13 |
| 2 | 11 | 11 | 14 |
| 3 | 12 | 13 | 15 |
| 4 | 12 | 12 | 18 |
| 5 | 13 | 12 | 18 |
| 6 | 13 | 13 | 16 |
| 7 | 12 | 12 | 16 |
| 8 | 13 | 14 | 16 |
| 9 | 11 | 14 | 12 |
| 10 | 13 | 13 | 11 |
| 11 | 11 | 14 | 13 |
| 12 | 12 | 12 | 12 |
| 13 | 12 | 14 | 10 |
| 14 | 9 | 16 | 11 |
| 15 | 11 | 14 | 13 |
| 16 | 13 | 14 | 12 |
| 17 | 7 | 16 | 11 |
| 18 | 9 | 15 | 12 |
| 19 | 12 |  | 14 |
| 20 | 11 |  | 13 |
| 21 | 12 |  | 12 |
| 22 | 11 |  | 14 |
| 23 | 13 |  | 12 |
| 24 | 11 |  | 13 |
| 25 | 11 |  | 12 |
| 26 | 9 |  | 12 |
| 27 | 9 |  | 12 |
| 28 | 13 |  | 15 |
| 29 | 12 |  | 12 |
| 30 | 13 |  | 13 |
| 31 |  |  | 14 |
| 32 |  |  | 12 |
| 33 |  |  | 13 |
| 34 |  |  | 14 |
| 35 |  |  | 12 |
